# Chloe’s Law: A Powerful Legislative Movement Challenging a Core Ethical Norm of Genetic Testing

**DOI:** 10.1371/journal.pbio.1002219

**Published:** 2015-08-06

**Authors:** Arthur L. Caplan

**Affiliations:** Director, Division of Medical Ethics, NYU Langone Medical Center, New York, New York, United States of America

## Abstract

Since the early 1970s, the ethical norm governing counselors involved in testing and screening for genetic conditions related to reproduction has been strict neutrality. Counseling about reproductive genetics was to be patient centered but nondirective. Many advocates for people with Down syndrome believe that high abortion rates following a diagnosis of this condition show an unfounded bias against those with Down syndrome. These advocates have succeeded in enacting federal and state legislation that requires women who receive a prenatal diagnosis of Down syndrome to receive positive information about the condition, thereby ending the nominal goal of value-neutral counseling and setting the stage for further normative shifts in clinical reproductive genetics as counseling expands because of cell-free testing.

## Abstract 2

This Perspective discusses a quiet revolution that is taking place in reproductive genetic counseling. Neutrality is under attack; advocates for persons with Down syndrome have succeeded in enacting legislation that requires women who receive a prenatal diagnosis of Down syndrome to also receive positive information about the condition, thereby ending value-neutral counseling.

This Perspective is part of the “Where Next?” Series.

## Introduction

Rates of abortion associated with a diagnosis of Down syndrome have been high for many decades. The recognition of this trend has led some families and supporters of people with Down syndrome to push for the enactment of a little-noticed law in Pennsylvania and many other states, the Down Syndrome Information Act, as well as a federal law, the Prenatally and Postnatally Diagnosed Conditions Awareness Act. The laws and the moral indignation that fueled their passage merit more attention and discussion.

The push for this legislation comes from an unlikely alliance of pro-life, pro-choice, and disability activists who argue that Down syndrome has been unfairly stigmatized. They also doubt that the norm of value neutrality is adequate to the task of informing women and their partners about how to think about Down syndrome. Yet, this relatively little-noticed drive to legislate against neutrality counseling may have far-reaching consequences, not only for testing for Down syndrome, but also for what information future patients may receive about other genetic conditions in the rapidly evolving field of reproductive genetic testing.

The Pennsylvania law, a version of the Down Syndrome Prenatal Education Act [1], known also as Chloe’s Law, was enacted in 2014. It is named after 11-year-old Chloe Kondrich, who has Down syndrome. Chloe’s dad was horrified to learn that the vast majority of mothers choose abortion if they learn their fetus is at risk for Down syndrome. He pushed the Pennsylvania state legislature to enact a law that requires the Department of Health to make available “up-to-date, evidence-based information about Down syndrome that has been reviewed by medical experts and national Down syndrome organizations,” including information on treatment options, support services, hotlines specific to Down syndrome, relevant resource centers, clearinghouses, and national and local Down syndrome organizations [1,2].

Down syndrome is the most common genetic disease in the United States. About one in every 700 babies is born with the condition [3]. The syndrome is associated with a distinctive facial appearance, some degree of cognitive impairment that can range from mild to severe, and a high incidence of other physiological problems such as heart defects, hearing impairment, and immune disorders.

Prenatal screening for Down syndrome using amniocentesis has been available since the early 1970s. Testing and counseling for Down syndrome became very common in the 1980s. Since amniocentesis can cause spontaneous abortion, for many years the reproductive genetics community emphasized limiting testing to women over the age of 35 who were at greater risk for fetal aneuploidy [4].

The ethical norm governing genetic counselors involved in testing and screening for genetic conditions related to reproduction has been strict neutrality since the early 1970s [5]: doctors and counselors were obligated to simply provide people with information to help them make decisions without guiding them toward a particular decision [4,5]. What patients do with test results in terms of deciding whether to have a child or to continue a pregnancy after a diagnosis of Down syndrome, counselors were taught, is strictly up to them. Whether the ethic of nondirective neutrality is or even could be adhered to has long been controversial [5,6], but it has been and remains the ethical aspiration for genetic counselors in the US and many other nations.

When it comes to testing for Down syndrome, the impact of genetic testing and counseling is clear—abortions. The United Kingdom National Down Syndrome Cytogenetic Register (NDSCR) reports that in 2012 92% of women with a positive prenatal diagnosis terminated the pregnancy [7]. Similar numbers prevail in the US [8]. Women make the same choice in other nations with readily available genetic testing [9]. Whether these high percentages are the result of cultural views that disparage this disability, the subtle shading of information by counselors against persons with Down syndrome, or worries on the part of parents about what Down syndrome means for the quality of life of their child or themselves is not clear. That genetic testing leads year after year to large numbers of abortions is.

Genetic testing explains why there are fewer children and adults around today in America with Down syndrome than might be expected. More women are having babies at older ages, which in the US should have produced a 25% increase over the past two decades in children with Down syndrome, which has not occurred [8].

What the Pennsylvania law and other similar laws enacted in eight states by the end of 2014 do is make sure that a woman who has undergone genetic testing for Down syndrome hears about the positive outcomes that are associated with having a child with this syndrome. Only positive information is mandated. This is because those promoting legislation believe, based on the abortion statistics, that parents of prospective children with Down syndrome do not receive sufficient and accurate information about the condition. They see the legislation as pro-information and, thus, pro–patient autonomy, although, if abortion rates did not change in states with such laws, it is fairly certain the legislation would be seen by many pro-life and disability proponents as a failure.

Legislation like Chloe’s law overturns the long-standing foundational ethical norm of genetic testing and counseling—neutrality in the provision of information [5]. Whether the frequent choice of abortion is due to a lack of information or simply reflects patient values is not clear. However, federal and state laws mandating a positive spin about Down open the door to what is sure to become a very heated debate about who should counsel and how to describe and discuss disabilities and differences as genetic testing becomes easier and safer to do using cell-free fetal DNA testing.

Chloe’s law is hardly value neutral. It is deliberately positive about Down syndrome. It (and other similar legislation that has been enacted in other states) seeks to spin the message given by doctors and counselors about Down syndrome in a particular direction. Kids with Down syndrome may have issues, the law concedes, but medical advances, devoted parenting, and societal resources can help ameliorate them. The associations of parents with children who have Down syndrome to which the law directs women who receive a prenatal diagnosis are made up of parents who are proud of their choice to have a child with Down syndrome, eager to fight for resources for opportunities and recognition for them, and keen to share stories of the positive value their child has had on their lives and those around them.

Chloe’s law seeks to shift the default of the ethics of genetic counseling about a condition that for decades has led many women to choose to end their pregnancies to a pronatalist stance. It is the first of what may become many other efforts to insist that those involved in genetic testing, screening, and counseling move away from nominal ethical neutrality to a more disability-friendly normative message.

With the imminent onset of a newer generation of fetal genetic testing, prenatal genetic testing is likely to become the standard of care for any pregnant woman, not just those deemed to be at high risk of having a baby with Down syndrome because of age. Recently, the results of a large, prospective, multicenter, blinded study were published, demonstrating that cell-free DNA testing, involving only a blood draw, for risk assessment of trisomy 21 (Down syndrome) outperforms combined first trimester screening in the general pregnancy population [10]. This means that testing will soon become routine for all pregnant women, regardless of age. It also means that genetic counseling will not be done by genetic counselors—there are simply too few of them. And using this technique, as genetic analysis improves, more and more conditions, traits, and differences will become detectable, thereby opening the door to the question of who will say what and with what ethical valence about why certain conditions are being tested for, what a positive test “means,” and what “risk” means in the context of deciding whether to continue a pregnancy.

Chloe’s law and laws like it have not yet had much impact on discussions of the ethics of genetic testing and counseling. Yet, this type of legislation may well turn out to be one of the most important events in the history of genetic testing and counseling. Value neutrality is highly unlikely to survive much longer as the appropriate or accepted stance for counseling and informing patients and their partners about the need for or results of genetic testing. Disability and pro-life groups will want information that puts disability in a positive light and abortion in a negative light to become a part of all counseling. Yet, mandating what a person seeking genetic testing needs to hear requires much more empirical information about what biases they bring to testing, what biases those doing counseling have, and whether “positive” information ought to be required or merely available depending on the patient’s choice. State laws such as Chloe’s law reveal the divide that exists between the rapid expansion in scope and availability of genetic testing and the lack of consensus regarding the ethical norms that ought to govern genetic counseling. All those involved in testing and counseling say they respect and value patient autonomy. But it is doubtful that neutrality should or will suffice to guide counseling as clinical testing rapidly expands.
